# Deconstructing tumor heterogeneity: the stromal perspective

**DOI:** 10.18632/oncotarget.27736

**Published:** 2020-10-06

**Authors:** Renee E. Vickman, Douglas V. Faget, Philip Beachy, David Beebe, Neil A. Bhowmick, Edna Cukierman, Wu-Min Deng, James G. Granneman, Jeffrey Hildesheim, Raghu Kalluri, Ken S. Lau, Ernst Lengyel, Joakim Lundeberg, Jorge Moscat, Peter S. Nelson, Kristian Pietras, Katerina Politi, Ellen Puré, Ruth Scherz-Shouval, Mara H. Sherman, David Tuveson, Ashani T. Weeraratna, Richard M. White, Melissa H. Wong, Elisa C. Woodhouse, Ying Zheng, Simon W. Hayward, Sheila A. Stewart

**Affiliations:** ^1^Department of Surgery, NorthShore University HealthSystem, Evanston, IL, USA; ^2^Department of Cell Biology and Physiology, Washington University in St. Louis, St. Louis, MO, USA; ^3^Department of Developmental Biology, Stanford University School of Medicine, Stanford, CA, USA; ^4^Department of Biomedical Engineering, University of Wisconsin – Madison, Madison, WI, USA; ^5^Department of Medicine, Cedars-Sinai Medical Center, Los Angeles, CA, USA; ^6^Department of Cancer Biology, Marvin and Concetta Greenberg Pancreatic Cancer Institute, Fox Chase Cancer Center, Temple Health, Philadelphia, PA, USA; ^7^Department of Biochemistry and Molecular Biology, Tulane University School of Medicine, New Orleans, LA, USA; ^8^Department of Molecular Medicine and Genetics, Wayne State University, Detroit, MI, USA; ^9^National Cancer Institute, Bethesda, MD, USA; ^10^Department of Cancer Biology, MD Anderson Cancer Center, Houston, TX, USA; ^11^Department of Cell and Developmental Biology, Vanderbilt University School of Medicine, Nashville, TN, USA; ^12^Department of Obstetrics and Gynecology, University of Chicago, Chicago, IL, USA; ^13^SciLifeLab, Department of Gene Technology, KTH Royal Institute of Technology, Stockholm, Sweden; ^14^Weill Cornell Medicine, Rockefeller University Campus, New York, NY, USA; ^15^Department of Medicine, University of Washington, Seattle, WA, USA; ^16^Department of Laboratory Medicine, Lund University, Lund, Sweden; ^17^Department of Pathology, Yale University School of Medicine, New Haven, CT, USA; ^18^Department of Biomedical Sciences, University of Pennsylvania, Philidelphia, PA, USA; ^19^Department of Biomolecular Sciences, The Weizmann Institute of Science, Rehovot, Israel; ^20^Department of Cell, Developmental and Cancer Biology, Oregon Health and Science University, Portland, OR, USA; ^21^Cold Spring Harbor Laboratory, Cold Spring Harbor, NY, USA; ^22^Sidney Kimmel Cancer Center, Johns Hopkins School of Medicine, Baltimore, MD, USA; ^23^Memorial Sloan Kettering Cancer Center, Weill Cornell Medical College, New York, NY, USA; ^24^Department of Bioengineering, University of Washington, Seattle, WA, USA; ^*^These authors contributed equally to this work; ^#^Workshop co-chairs

**Keywords:** tumor microenvironment, stromal heterogeneity, extracellular matrix, cellular plasticity, therapy resistance

## Abstract

Significant advances have been made towards understanding the role of immune cell-tumor interplay in either suppressing or promoting tumor growth, progression, and recurrence, however, the roles of additional stromal elements, cell types and/or cell states remain ill-defined. The overarching goal of this NCI-sponsored workshop was to highlight and integrate the critical functions of non-immune stromal components in regulating tumor heterogeneity and its impact on tumor initiation, progression, and resistance to therapy. The workshop explored the opposing roles of tumor supportive *versus* suppressive stroma and how cellular composition and function may be altered during disease progression. It also highlighted microenvironment-centered mechanisms dictating indolence or aggressiveness of early lesions and how spatial geography impacts stromal attributes and function. The prognostic and therapeutic implications as well as potential vulnerabilities within the heterogeneous tumor microenvironment were also discussed. These broad topics were included in this workshop as an effort to identify current challenges and knowledge gaps in the field.

## INTRODUCTION

The last two decades have seen significant gains in unveiling the crosstalk between cancer cells and the stroma within which they reside. This complex mixture of interacting cells can be considered as a dynamic multidimensional ecosystem that regulates tumor growth, inherent and acquired plasticity/heterogeneity, invasion, and metastasis. The expansion of cancer research beyond a cell autonomous-based model has enabled the identification and characterization of a wide range of cell types including fibroblasts, immune, endothelial, neuronal, and specialized mesenchymal cells that regulate the formation of a tumor-permissive and therapy-resistant environment. In addition, a deeper appreciation for the role that the extracellular matrix (ECM) plays in this ecosystem has emerged. Despite these advances, much of the scientific focus has remained primarily centered on the tumor cells and on driver mutations that dictate stromal reprogramming to support tumor growth. These include oncogene-mediated tumor cell proliferation, reactive oxygen species (ROS)/hypoxia-induced regulation of the endothelium, inflammation-associated angiogenesis and immune-suppression, and metabolic reprogramming of stromal fibroblasts and adipocytes to offset tumor cell nutrient deprivation [1–5]. Studies in these areas have collectively uncovered numerous mechanisms by which cancer cells manipulate the microenvironment to support tumor growth. However, many aspects of the biological alterations that arise in stromal cell types or in the extracellular matrix components that may drive cancer initiation or progression remain poorly understood.

The traditional view of driver mutations within incipient tumor cells instructing each step of tumorigenesis has influenced basic cancer researchers and drug developers alike to focus on genetically and molecularly profiling tumor cell autonomous mechanisms for targetable vulnerabilities. The foundational principle of “a tumor cell as an organizer” [6] also gave rise to precision medicine as a promising blueprint and path forward to more effective mutation- or target-specific therapeutic strategies. Unfortunately, only modest improvements in clinical outcomes have resulted from these approaches. Aggressive tumor phenotypes resulting from targeted therapy, such as small cell lung cancers and neuroendocrine prostate tumors following EGFR- and AR-targeted inhibition [7, 8], respectively, prompted the community to conclude that narrow tumor cell and mutational status-centric approaches alone are unlikely to succeed. The realization that successful combination therapies in broad tumor types required an understanding of the complexity of tumor-tumor microenvironment (TME) dynamics and heterogeneity led to the development of immune-oncology and checkpoint inhibitors. The availability of research tools, experimental models, and clinical reagents in the field of immunology has enabled the rapid rise and validation of novel mechanism-based approaches to modulate the immune microenvironment.

The delivery of durable remission induced by anti-programmed cell death protein 1 (PD-1)/programmed death-ligand 1 (PD-L1) and cytotoxic T-lymphocyte-associated protein 4 (CTLA-4) immune checkpoint inhibitors in a subset of cancer types and patients has motivated many basic and translational investigators to focus their attention and resources towards studying the role of the immune cell-tumor interplay in cancer growth, progression, and recurrence [9–11]. However, only a subset of patients benefit from immunotherapies and little is known about the contribution of non-immune stromal cell types and extracellular matrix components to these processes, and even less is known about how these stromal functions and interactions differ throughout each stage of disease progression [11, 12]. Nevertheless, these successes raise the possibility that further disease altering targets reside in the TME, awaiting discovery.

Limited, yet pivotal studies demonstrating the importance of non-immune stromal components in shaping the fate of tumors exist, but remain poorly understood and underpowered. Examples of work that must be expanded include understanding the role of non-immune stroma in regulating immune cell recruitment to tumor sites, driving chronic inflammation-induced DNA damage in preneoplasia, characterizing and tracing different carcinoma-associated fibroblasts (CAF) functional subtypes and their roles in different states as both promoters and suppressors of tumor progression, and the direct causal relationship between acellular stroma, ECM remodeling and/or age-related senescence-associated secretory phenotype (SASP) in inducing a field-effect conducive to malignant conversion.

An ongoing challenge in studying these non-immune stromal programs is that the models are still limited. Appropriate and highly specific Cre-drivers that allow for dynamic fate mapping of these cells are often lacking. Limited studies have genetically manipulated these stromal cell types in an unbiased manner using shRNA and/or CRISPR approaches. This is essential for discovering new functional aspects of stroma-tumor crosstalk to move it from correlation to causality. Current models that will enable these discoveries include more rapid mouse transgenic models, complex organoid models, as well as zebrafish and flies, the latter of which are particularly amenable to screens of this sort.

Expanding the notion that stroma, along with the tumor cells, co-organizes all stages of tumorigenesis will lay the foundation for a broad spatial and temporal understanding of the complete TME from initiation through metastasis. In order to assess the challenges associated with this goal, the NCI’s Division of Cancer Biology sponsored a workshop that built upon the advances in the immune-oncology realm and emphasized non-immune stromal components. The workshop highlighted the critical functions of the non-immune stromal cell types and ECM in regulating heterogeneity and tumor cell fate throughout initiation, progression, and therapy resistance. The workshop not only explored the multifaceted role of stroma in suppressing and/or supporting tumorigenesis in early and advanced stage cancers, but also considered emerging themes of stromal plasticity and position-dependent functions of stromal subtypes in driving disease progression, resistance to therapy, and as possible clinical targets of vulnerability. These workshop topics were consolidated within five themes that addressed key scientific and technical challenges related to 1) stromal-centric arbiters of tumor progression and suppression; 2) the unique biology of early lesions in regulating indolence versus aggressiveness; 3) stromal plasticity and its role in regulating the fate of the tumor mass; 4) the significance of stromal cell geography and architecture in cancer; and 5) clinical implications of microenvironmental heterogeneity.

### Stromal-centric arbiters of tumor progression and suppression

Stroma contributes to both suppressing and promoting tumor progression, yet what cell subtypes, ECM components, and underlying mechanisms regulate tumor fate in varying contexts, including aging, genetic background, and systemic comorbidities, remain ill-defined. Studies conducted on fibroblasts and CAFs as a major stromal element have contributed significantly in the recent past to characterizing the heterogeneity of the microenvironment and important leads are beginning to emerge that elucidate distinct functions of different subtypes in these diametrically opposing functions. In the first talk of the session, Raghu Kalluri highlighted the functional diversity of tumor-restraining (αSMA+) and tumor-promoting (FAP+) CAF subtypes in a mouse model of pancreatic ductal adenocarcinoma (PDAC). αSMA+ CAFs predominantly modulate ECM production, facilitate cell-ECM adhesion, and regulate adaptive immunity [13].

Simon Hayward shifted the focus to human prostate cancer models and provided evidence of CAF subtypes with distinct expression signatures revealed by single cell RNA-seq analysis. CAF clusters differ in their chemokine/cytokine profiles, leading to cluster-specific effects on the microenvironment – such as macrophage recruitment via high CCL2 expressing CAFs or modulation of inflammatory cells by high CXCL12-expressing CAFs. Specific populations of recruited populations (depending on the CAF subtype) can produce distinct factors that will shape the tumor and/or microenvironment in distinct ways – e.g., macrophage-derived pro-tumorigenic factors, recruitment of other immune cell types that promote the M2-macrophage phenotype [14].

In her presentation, Ellen Puré focused on reactive CAFs expressing fibroblast activation protein positive (FAP+) in mouse PDAC. In contrast to normal stromal cells that maintain epithelial integrity and confer tumor resistance, FAP+ fibroblasts have immunosuppressive and tumor promoting properties. Indeed, both the Puré and Kalluri laboratories have provided evidence that human PDAC patients with FAP+ fibroblasts have decreased survival compared to αSMA+ fibroblasts [13, 15]. FAP+ fibroblasts display increased ECM alignment [16] and synthesis, yet lower contractility and ECM crosslinking, while displaying higher levels of paracrine growth factors and inflammatory gene expression profiles [17]. Mechanistically, Puré highlighted a novel ECM-informed stromagenic switch – regulated by multiple factors including substratum stiffness, PDGF, TGFβ, sonic hedgehog, and osteopontin – that contributes to CAF heterogeneity and is a key component of the transition from a tumor-resistant to a tumor-permissive microenvironment [18].

David Tuveson discussed findings from his team involving the identification of additional CAF subtypes classified as either myogenic myCAFs, inflammatory iCAFs, or the most recently isolated and characterized antigen presenting apCAFs that express MHC class II and CD74 and are capable of activating CD4+ T cells, but lack other immune related genes including classical co-stimulatory molecules [19, 20]. Studies by his group using 3D organoid and mouse models suggest that CAF heterogeneity is in part driven by IL-1β and TGFβ antagonism, resulting in iCAFs and myCAFs that either promote or inhibit tumor growth, respectively [21]. Single-cell mRNA-seq of human PDAC also putatively identified these two CAF subpopulations [19]. He further highlighted the inherent, position-dependent plasticity between CAF subtypes, suggesting these are functional fibroblastic states, as opposed to static fibroblast types.

The topic of cellular plasticity was expanded further by Ashani Weeraratna in her discussions of the aging stroma in a mouse model of melanoma. She summarized data that suggest age-associated events contribute to altering the function of CAFs in a tissue-specific manner and with important implications to not just tumorigenesis and metastasis but also therapeutic outcomes. For instance, aged lung fibroblasts (but not aged skin fibroblasts from human donors) drive proliferation via induction of canonical Wnt signaling [22]. In contrast, aged skin fibroblasts exhibit enhanced sFRP2-driven non-canonical Wnt signaling that triggers a phenotypic switch to a non-proliferative, yet highly invasive and BRAFi-resistant mesenchymal state [22]. This BRAFi/MEKi resistance is further propagated by the secretion of lipids by aged fibroblasts, which drive metabolic changes in melanoma cells in aged microenvironment (Alicea, Rebecca et al. 2020). Targeting these age-related changes overcame therapy resistance in animal models, suggesting that the TME may provide a rich source of targetable moieties.

Fibroblasts and other stromal cell types in the tumor microenvironment acquire pro- and/or anti-tumorigenic phenotypes that impact carcinoma growth. Research in this area has identified heterogeneity in CAF marker expression and function, altered functions of fibroblasts based on tissue source, and plasticity of fibroblasts among cell states. A greater understanding of this functional heterogeneity and determining the degree of plasticity in other stromal cell types will elucidate novel mechanisms of tumor promotion and/or suppression, which may have therapeutic potential.

### Biology of early lesions: indolence *versus* aggressiveness

It has become increasingly clear that the TME changes throughout disease progression, but the involvement of stromal cells is primarily studied with regard to established and more advanced stages of cancer (e.g., metastasis). The contribution of stromal cells and the ECM to tumor initiation has not been widely studied, but some research in this area using diverse approaches has opened exciting avenues for further investigation.

Wu-Min Deng actively investigates tissue environments which are susceptible or resistant to tumor formation using a *Drosophila* model. He described that, upon knockdown of a tumor suppressor gene *scrib*, specific regions within developing fly tissues would reject tumor growth while other regions would allow tumor growth. This identified “tumor hotspots” in which JAK/STAT signaling was determined to be elevated [23, 24]. Further analysis of transition cells of imaginal rings revealed that notch signaling drives mitosis in these polyploid cells [24]. These studies provide evidence that signaling events within specific microenvironments can enable tumor formation. Understanding the “tumor hotspot” and “tumor coldspot” microenvironments and what controls the definition of these regions may aid in cancer prevention or have therapeutic potential.

Philip Beachy highlighted work from his group demonstrating stromal suppression of growth in tumors of endodermal origin. In bladder cancer, tumors are almost completely clonal in origin, and using a mouse bladder cancer model his group determined that sonic hedgehog (Shh)-expressing cells act as long-term stem cells that regenerate the bladder epithelium via Wnt signaling [25, 26]. Precancerous bladder epithelium is still Shh+ but expression is lost in invasive carcinoma [26]. The transition to invasive carcinoma may be linked to loss of bone morphogenic proteins (BMP) in the adjacent stromal tissue, since BMP treatment promotes differentiation and blocks invasion in a mouse model of bladder cancer [26].

Another approach to investigating stromal involvement in early cancer lesions was described by Mara Sherman, who studies stromal evolution with pancreatic stellate cells (PSCs). Work by her group is focused on understanding the origins of fibroblasts within the TME in mouse PDAC. PSCs give rise to the PDAC CAF phenotype, but other non-PSC-derived CAFs exist as well. Using known markers of fibroblasts, such as desmin and podoplanin (PDPN), she indicated that some PDAC CAFs were PSC-derived while other CAFs were PDPN-positive, non-PSC-derived CAFs [27]. She provided new evidence that activated PSCs contain less lipid droplets compared to pre-activated, and fatty acid binding protein 4 (FABP4) may be used as a marker for PSC-derived CAFs in PDAC tissues [28]. However, more work is needed to identify reliable markers for various CAF subsets to further understand this heterogeneity.

The discussion of this session was expanded further by Ken Lau, who uses computational modeling in colon cancer to identify CAF-cancer cell interactions that drive tumor progression [29]. His studies indicate that CAFs dominate the signaling networks of cell surface receptor-ligand interactions, specifically during epithelial-mesenchymal transition (EMT) and in inflammatory nodules (unpublished data). Noninvasive colon cancer cells may also express EMT markers, so these are not unique to metastatic cells. His laboratory identified that PDGFRα+ CAFs are adjacent to cancer cells in colon cancer tissue specimens while αSMA+ myofibroblasts are more distant, indicating potentially unique functions of these fibroblast subsets as the disease progresses and underscoring the importance of geographic location within a developing and metastasizing tumor (unpublished data). Research by the Lau group and others in this session highlighted the exciting opportunities for discovery in the area of early-stage tumor biology. Further investigation of stromal evolution is necessary to understand the biological importance to tumor formation at different stages as well as to evaluate stage-specific therapeutic implications in these diseases.

### Stromal plasticity and communication

Complete knowledge of the TME requires an understanding of the many cell types and interactions within it. Unfortunately, the complexity of these interactions makes this work technically and conceptually difficult. Some of these interactions cannot be adequately studied because appropriate tools have not yet been developed. Thus, one of the objectives of this meeting was to discuss interactions between all cell types within the TME, including fibroblasts, adipocytes, immune cells, endothelial cells, nerves, normal epithelial cells, and cancer cells, as well as to discuss current limitations associated with researching intercellular communication.

Throughout the last decade, our understanding of cancer-immune cell interactions has been significantly advanced and has even led to successful new therapeutic strategies. PD-L1 targeting is one example of recently discovered therapeutic options, but clinical applications of PD-1/PD-L1 therapies have identified limitations, specifically by driving resistance mechanisms in tumors [11]. Current work by Jorge Moscat’s research group has shown that PD-L1 treatment works by revitalizing CD8+ T cell infiltration in a mouse model of serrated colorectal cancer, but only in young mice in which tumors have not yet developed a reactive stroma [30]. In older mice with highly desmoplastic tumors, PD-L1 treatment does not restore CD8+ T cells and consequently has no curative activity. This finding suggests that dual stromal activation impairs immune checkpoint blockade therapy, which was demonstrated to be the case by Moscat’s lab by targeting of TGFβ, which enhanced PD-L1 targeting efficiency [30]. Furthermore, analysis of how stromal cells may impair PD-L1 therapy suggests that CAF can block antigen presentation. Consideration of additional stromal contributors in this context as well as systemic factors, such as age, will elucidate superior therapeutic options.

While the role of some immune cell populations, notably T cells and tumor-associated macrophages, have been widely studied in the context of the TME, this is an active area of research in which novel findings continue to evolve our understanding of immune cell-tumor cell dynamics and their potential implications in shaping tumor evolution. Melissa Wong and her group have recently characterized a cell fusion of macrophages with cancer cells [31]. These cancer hybrid cells acquire macrophage gene expression profiles and retain macrophage behavior, but are also capable of initiating tumors and can be detected in peripheral blood of patients [31, 32]. While most studies involving circulating tumor cells (CTCs) use cytokeratin+CD45- as the schema for detecting CTCs, Wong’s group discovered that hybrid cells that are cytokeratin+CD45+ make up more than 90% of circulating cancer cells [32]. This discovery suggests that fusogenic events between CD45- epithelial cells and CD45+ macrophages are at play in the TME in addition to more widely appreciated cell-cell interactions and that these events may impact tumor progression.

Significant efforts are currently in progress to target immune cells, so discussions at this meeting put greater emphasis on other cell types in the microenvironment that have not been so widely studied. Adipose tissue, for instance, is very dynamic and may serve as a nutrient source for cancer cells. Ernst Lengyel works to understand the microenvironmental contributions to human ovarian cancer metastasis and the metabolic co-dependencies between cancer and stromal cells. Metastasis to the omentum, the common site in this disease, leads to the localized disappearance of adipose tissue in the affected area. Dr. Lengyel’s group has provided evidence that this loss is likely due to the contributions of adipose tissue lipids and FABP4 to the tumor cells [33]. Ovarian cancer cells become loaded with lipid droplets after co-culture with adipocytes [34]. Similarly, the Hayward lab shows that prostate CAFs also maintain increased lipid droplet density compared to normal prostate fibroblasts, which aid in prostate cancer cell growth *in vitro* [35, 36]. Conversely, activation of primary PSCs yielded significantly less lipid droplets compared to pre-activated PSCs, but nevertheless suggest a link between stroma-derived lipids and tumor metabolism and growth [28]. However, the source of these lipids, the role they play in individual stromal cell types, and whether these differ in unique cancer types remain to be seen.

To further elucidate the role of lipids and adipose tissue in the TME, James Granneman’s laboratory investigates adipogenic niches and the stromal/immune cells that are key to adipose tissue maintenance, remodeling, and may play a role in tumor progression. scRNA-seq of mouse adipose tissue by his group identified two major adipocyte stem cell (ASC) subpopulations, ASC1 and ASC2, in epididymal and inguinal white adipose tissue that express different collagens and trophic factors, such as neurotrophin 3 and bone morphogen 7 [37]. The ASC1 subpopulation expresses higher levels of PPARγ, a master regulator of adipogenesis, as well as the adipogenesis markers caveolin-1 and G0/G1 switch 2 – and appear to be primed for adipogenesis compared with ASC2 cells. The Granneman lab has begun to map the trajectory of adipose tissue in mouse cancer models. In these studies, tumor-bearing mammary fat pads underwent ASC population shifts from ASC2 to ASC1 in tumor-bearing mice as well as changes in macrophage expression patterns linked to inflammatory responses, such as increased Trem2 and Irf8 [37]. This work aims to discover how adipose tissue changes in cancer and mechanisms by which this evolution contributes to malignant progression.

Richard White further expanded the discussion of stromal-cancer cell interactions by discussing his work using zebrafish melanoma models. The zebrafish is ideal for this given the optical transparency of the *casper* strain, which allows for powerful *in vivo* imaging [38]. Melanomas are surrounded by two cell types organized into a distinct geography, with keratinocytes above the lesion and adipocytes below the lesion. Prior work from the White lab showed that adipocytes are major drivers of melanoma progression by donating fatty acids to the tumor cell [39], which is consistent with other work in ovarian cancer [40]. What is less understood is how the keratinocytes, which are normal epithelial components of skin, drive progression. Using a BRAFV600E induced transgenic melanoma model [41] and real-time *in vivo* imaging, the White lab found that tumor cells export a variety of extracellular vesicles such as melanosomes and exosomes into the seemingly normal keratinocytes. The recipient keratinocytes then undergo reprogramming and switch from a tumor suppressive to tumor promoting role. These reprogrammed keratinocytes exhibit an N-myc pluripotency signature and dramatic alterations in chromatin structure. Although the absolute number of keratinocytes that undergo such reprogramming is small, their importance in promoting melanoma progression highlights the need for a detailed understanding of the “geography” of tumor-stroma interactions [42].

In other cancer models, such as renal cell carcinoma, the environmental context of cancer epithelial cells drastically influences the invasion potential of epithelial tumor cells. David Beebe’s models that incorporate compartmentalized epithelial and endothelial duct-based organotypic platforms, indicate that incubation of tumor epithelium with normal endothelium promotes invasion of the endothelial cells. However, incubation of tumor epithelium with cancer associated endothelium only yields cancer cell invasion without endothelial invasion. The Beebe lab continues to lead the development of cell-cell interaction and microfluidic models for the study of unanswered biological questions [43, 44]. Use of these models and others will be essential to understanding the role of specific stromal cell types, intercellular communication, and the role of ECM components in the TME.

### The significance of stromal cell geography and architecture

The late Dr. Patricia Keely revolutionized the TME field by demonstrating that architectural organization of the ECM (including tumor cell distribution/localization) is fundamental to tumor cell-stromal cell interactions, metastasis, and response to therapy [45–48]. A stromal/ECM barrier or tumor cell location within the TME may not only create nutrient/growth factor gradients, but also serve as a source of exogenous physical forces that may reprogram tumor cells and potentially function as a barrier against cancer therapy and adaptive immune cell destruction. Stromal stiffness, for instance, can signal normal epithelial cells to adopt malignant phenotypes [49, 50], while dormant tumor cells have been suggested to resist chemotherapy by residing within the perivascular niche [51]. Therefore, a better understanding of tumor architecture and histology can provide important knowledge about how stromal cells organize themselves around the tumor cells and dictate their function/phenotype.

Despite recent technological advances, such as imaging mass cytometry and co-detection by indexing (CODEX) [52], histological analyses remain limited due to reliance on pre-existing antibodies. To overcome this limitation, Joakim Lundeberg has recently developed Spatial Transcriptomics (Visium from 10X Genomics, Inc.), a tool that combines tissue imaging with barcode array-based sequence transcriptomics. This tool allows for standard microscopy of fresh frozen sections to be coupled with spatial mRNA-seq, and ultimately visualization of imaging patterns, clonal tumor populations and stromal areas within intact tissues. Key findings include the identification of discrete stromal regions around prostate tumors that were nonresponsive to treatment [53]. As they expand the analysis, new fibroblast expression datasets under development will help identify CAFs of different origins and functions within the TME. The spatial interaction between stroma and various tumor subpopulations at the leading edge has recently been investigated in squamous cell carcinoma [54].

Next, Kristian Pietras showed that breast cancer CAFs from a mouse model are characterized by three distinct transcriptional programs, potentially reflecting unique spatial origins within the TME [55]. The largest CAF populations were enriched for angiogenic genes (vCAF) reflecting a perivascular origin. The second most abundant CAF population showed a significant increase in the expression of ECM-associated genes, suggesting tissue-resident fibroblasts as the source for this specific CAF subtype. The third, scarce CAF cluster was characterized by the expression of development-associated genes and were provisionally named developmental CAFs (dCAF). dCAFs show some overlapping gene expression with tumor epithelium, suggesting that dCAFs may originate from cancer cells that underwent EMT. The combination of Pietras’s CAF dataset with spatial transcriptomics may provide interesting data regarding the real spatial localization of these CAF subtypes within the TME.

Ruth Scherz-Shouval discussed transcriptomic data from a murine breast cancer model obtained using single-cell RNA-seq at different time points along tumor progression and metastasis. Her group identified two distinct CAF populations, each of which could be further dissected into subsets that change as tumors progress. These CAF subtypes, sCAF and pCAF, were characterized by high expression of S100A4 and PDPN, respectively, and found in both mouse and human breast tumors. Interestingly, a higher sCAF/pCAF ratio was indicative of better overall survival in human patients, suggesting that the sCAF population may play a protective role against tumor progression [56]. Identifying specific subsets of CAFs that are associated with better therapeutic response and survival will allow researchers to leverage the function of these CAFs and bring precision treatment to patients with tumor-permissive CAF subtypes within their TME. However, as the characterization of CAF subtypes advances, the field will need to put further effort into standardizing the nomenclature and markers of these unique CAF populations (i.e., lineages and functions). Doing so will help integrate the findings from different/independent research groups.

Katerina Politi shifted the focus to discuss how tumor intrinsic-targeted therapies are impacted by the organs affected by metastases. As mentioned above, these approaches have limited benefits, since tumors may, in part, undergo clonal selection upon targeted-therapy eventually culminating in a more aggressive tumor. For example, in EGFR-mutant lung tumors, resistance to first and second generation tyrosine kinase inhibitors (TKIs) is mostly driven by the emergence of a secondary T790M mutation in EGFR. To overcome this resistance, a third generation TKI, osimertinib, is currently used clinically, but patients can still develop resistance and many of the resistance mechanisms are poorly understood. To unravel the underlying mechanism, Politi and colleagues are investigating therapeutic responses in different metastatic sites. Early data support the idea that the TME location can impact sensitivity to TKIs. It is well known that different tumor sites can differentially impact tumor progression. Hence, the field needs to advance towards a better characterization of the molecular and cellular mechanisms that drive increased tumor progression in an organ and location-specific manner. Concomitantly, improved comprehension of the specific sites in the body that more potently restrict tumor cell proliferation could also provide valuable knowledge about stromal/tumor interactions that drive tumor progression.

The meeting also tackled the question of endothelial heterogeneity. Ying Zheng discussed the dynamic nature of the tumor microvasculature and how it changes during tumor progression. Zheng noted that many ongoing studies still work with specific cell lines that do not recapitulate the heterogeneity found in endothelial cells of different tissues. It is also important to consider the interactions of endothelial cells with their surroundings, which contain diverse cell types, ECM, and geometric complexities. Taking this into consideration, Zheng engineered a device that allows endothelial cells to reconstruct 3D vessel structures using fetal endothelial cells from different tissues. This technology aided in determining that endothelial cells with distinct tissue origins do indeed form vessels with diverse architecture and unique transcription profiles [57]. Studies regarding ECM influence on microvasculature architecture and gene expression are ongoing and will help elucidate the crosstalk between endothelial cells originating in different organs and their surroundings. Many questions still need to be addressed in order to completely understand microvasculature formation and function in tumors, including endothelial cell plasticity and how tumor cell or tissue location impacts endothelial cell behavior and microvasculature structure and function.

The present level of characterization of CAFs, adipocytes and endothelial cells has brought valuable, but less than comprehensive information about their function within the TME. Nevertheless, it will help develop a necessary expansion in the field to identify new opportunities for targeted therapies. Better understanding the role of the non-immune stroma in shaping the microenvironment prior to the appearance of a tumor is also pivotal to unravelling the mechanisms driving tumorigenesis. A key issue is the high degree of stromal cell plasticity driven by multiple environmental vectors, creating a challenge to working with them in an *ex vivo* setting. Another looming challenge is to define cell states *versus* subtypes, particularly in CAFs. Also, work toward identifying triggers of cellular plasticity *versus* a more permanent epigenetic state is needed. This work will lay the groundwork for the field to move more deeply into the mechanisms that drive changes in the TME and impact tumorigenesis.

### Prognostic and therapeutic implications of microenvironmental heterogeneity

During the final session, discussions were focused on the implications of current cancer therapy in the TME. Despite recent advances in targeted and immunotherapies, the core of cancer treatment is based on genotoxic agents, such as ionizing radiation and chemotherapy. Those agents exert systemic and local damage in non-tumor cells that consequently impact therapy-associated comorbidities and cancer recurrence. In this context, Sheila Stewart discussed her findings related to the impact of senescence in chemotherapy-induced bone loss, a common comorbidity in cancer patients. Using a genetic mouse model, her team found that elimination of senescent cells that arise after chemotherapy prevented bone loss [58]. In addition, targeting SASP pathways using pharmacological inhibition of p38MAPK/MK2 signaling also limited chemotherapy-induced side effects [58]. Senescence is not only triggered by chemotherapy but occurs normally in healthy individuals, as part of the aging process, and may directly impact tumorigenesis. Senescent fibroblasts upon SASP expression behave similarly to CAF regarding their ability to directly support tumor growth [59]. Stewart highlighted that limiting SASP signaling pathways reduces metastatic growth in a breast cancer setting. Her group found that inhibition of p38MAPK/MK2 signaling restrained metastatic growth in the bone and visceral organs, through tumor cell-independent mechanisms [60]. However, the stromal cells where p38MAPK/MK2 signaling exerts a deleterious role in metastatic growth remain to be elucidated.

Besides SASP, chemotherapy-induced genotoxic stress can trigger a DNA damage secretory program (DDSP) – very similar to SASP but not associated with cell cycle arrest and/or p16 signaling– releasing numerous inflammatory (IL-6/8), angiogenic (VEGF, CXCL1), mitogenic (amphiregulin), pro-EMT (HGF) and chemo-preventative factors. Focusing on the potential effects of genotoxic stress in the TME, Peter Nelson showed that human prostate fibroblasts upregulate numerous pro-tumorigenic factors upon a variety of genotoxic stimuli. Among the released factors, WNT ligands attenuate a cytotoxic chemotherapy effect *in vivo* when expressed in the prostate TME [61]. He highlighted the therapeutic potential of targeting these chemotherapy-induced factors, many of which are downstream of NF-κB and mTOR pathways. Despite the fact that NF-κB does not currently constitute a viable target, mTOR signaling can be targeted by many available drugs (e.g., rapamycin). Interestingly, fibroblasts that have been previously treated with rapamycin do not confer chemotherapy resistance to prostate cancer cells *in vitro* and *in vivo*. These data support ongoing clinical trials to assess whether rapamycin-induced inhibition of DDSP in prostate cancer patients under chemotherapy treatment can improve response. Other druggable DDSP pathways, including PARP inhibition to attenuate the DDSP and the NKG2D-MIC-MMP axis to eliminate damaged cells with immune-suppressive phenotypes, are also under consideration for future studies to identify new opportunities for stroma-targeted therapies [62].

Neil Bhowmick expanded on the stromal considerations for prostate cancer therapy by presenting findings that demonstrated chemotherapy-triggered crosstalk between CAFs and tumor cells. His team found that docetaxel induces mitophagy and ER stress in prostate tumor cells, leading to secretion of mitochondrial DNA (mtDNA) [63]. This results in TLR9 activation in CAFs and release of C3a, a complement protein. Interestingly, C3a-induced activation of the C3a receptor in prostate cancer cells drives docetaxel resistance in mouse models. mtDNA was also found in the plasma of docetaxel-treated patients supporting the hypothesis that therapy resistance in prostate cancer patients may occur by this crosstalk [63]. In mouse models, combination therapy of docetaxel with a C3aR inhibitor showed increased efficacy in limiting tumor growth when compared to docetaxel alone. Future studies to unravel stromal-mediated chemotherapy resistance mechanisms may support the development of stroma-targeted therapies that improve current chemotherapy response in different types of cancer.

Finally, Edna Cukierman discussed the stromal heterogeneity found in the desmoplastic environment of pancreatic tumors. Her group found that fibroblastic cell function depends on the fibroblastic ECM. She showed that the desmoplastic ECM sustains the expression of Netrin G1 in PDAC CAFs while tumor adjacent fibroblasts cultured in “normal” ECM do not express this protein. Netrin G1 is a glycosylphosphatidylinositol-anchored protein known for its glutamatergic pre-synaptic function. Interestingly, the Netrin G1 binding partner, NGL1, is overexpressed in pancreatic cancer cells, suggesting a possible crosstalk between CAFs and tumor cells through Netrin G1/NGL1 signaling axis [64]. Further investigation revealed that a lack of Netrin G1 in fibroblasts or lack of NGL1 in pancreatic cancer cells severely affected the ability of the latter to survive upon nutrient starvation. In addition, Netrin G1 expression in CAFs, through downstream p38MAPK activation, was found to be required to suppress NK cell-mediated cytotoxicity in the same model. Further, her team revealed that high expression levels of stromal Netrin G1 inversely correlate with PDAC patient overall survival. Taken together, the presented data indicate that Netrin G1+ CAFs provide better PDAC cell survival in nutrient-deprived environments, and means to escape from the immune system. Notably, it highlights the multiple roles CAFs play during tumor progression. As the field moves forward, TME researchers can build upon these studies that show stroma support tumor growth and resistance to treatment and/or stress in multiple environments.

### Outstanding questions

This meeting fostered discussions of the needs and limitations of research currently focused on the components of the TME. Participating individuals are leading research programs to identify novel cell types, pathways of cellular interactions, model systems suitable for TME research questions, and to unravel heterogeneous cell populations. However, investigation of the TME components – and how they evolve throughout tumor progression, their role as drivers or suppressors of tumorigenesis, malignancy and/or partner with the tumor mass as co-organizers in tumor growth and respond to current therapeutics – remains limited. While progress has been made by several research programs in identifying novel CAF subpopulations, and initial traction is beginning to emerge with endothelial cells and adipocytes, standardized functional and molecular definitions for fibroblast subtypes (and other non-immune cells) do not yet exist. Tools to capture stromal heterogeneity are limited due to variable isolation techniques and the loss of cellular and microenvironmental context. Even with scRNA-seq data acquired to-date, lack of uniform analysis pipelines makes full utilization of the biological implications of this data difficult.

It would also be beneficial to create an accessible catalog of all cell types, spatial context, mechanical properties, and events that regulate them such that the field may begin to interpret what constitutes a supportive or resistant tumor stroma – or even sub-stromal compartments such as the perivascular niche that may uniquely protect tumor cells relative to other stromal regions. Similarly, it is not yet understood when a stroma becomes supportive of the tumorigenic process, when a suppressive stroma is lost or reprogrammed, and what determines this “stromagenic switch.” This includes the contribution of lifestyle factors, aging, and drug treatments that are likely to not only impact traditionally (and narrowly) defined tissue-specific microenvironments but also more broadly if the microenvironment is expanded to systemic effects as well. Another consideration is the changes in stroma from early (pre-neoplastic) disease through metastatic disease, which likely abide by stage-specific regulatory networks and yield unique changes for each cancer type. While many microfluidic or other engineered model systems are being developed to address the interactions of multiple cell types, development of new models may be necessary to address these fundamental questions.

Cells from multiple origins may perform similar functions. We have discussed CAF heterogeneity broadly in terms of function and marker expression, but these cells may be derived from a variety of sources, including bone marrow-derived mesenchymal stem cells, adipocytes, or even cancer cells which have undergone EMT [5]. Despite distinct origins, these precursors may converge into similar CAF functions, reinforcing the importance of key stromal cell functions in the TME.

Finally, we need to determine what therapeutic opportunities exist within the TME that may improve the current standard-of-care for each cancer type and disease stage. Understanding some of the questions outlined above may unveil new therapeutic strategies, but widely useful platforms to test novel strategies both in primary and metastatic disease settings are also necessary, particularly because a response in the primary setting does not dictate a response in the metastatic setting. Likewise, established tumors are not one and the same as an early lesion, and are therefore unlikely to follow the same set of “biological rules.” Since the TME is complex and evolves with disease progression, there are many opportunities for discovery. Ultimately, a greater research effort in tumor stroma will lead to precision medicine with optimal therapeutic intervention and better patient outcomes.
